# Dear Readers, Authors, Reviewers and Editorial Board Members of *Advances in Cognitive Psychology*

**DOI:** 10.5709/acp-0201-z

**Published:** 2017-03-31

## Abstract

In this first newsletter of 2017, we wanted to inform you about important developments concerning our journal and the performance of the journal in 2016. We also would like to draw your attention to the fourth issue of Advances in Cognitive Psychology of 2016, which is a special issue that includes several contributions to the Neuronus conference that was held in Kraków in 2015. The current newsletter will also be included in the first issue of 2017.

## Important developments

At the end of 2016, it became clear that our journal was in need of further financial support as the University of Finance and Management in Warsaw was no longer able to cover the costs. Multiple ways were considered as to how to continue with the journal. A first idea, which was thoroughly discussed with several of our editorial board members, was to examine whether we would be able to obtain sufficient financial support from scientific institutions (i.e., universities and professional associations) to continue with our free-of-cost open-access publishing policy. We are grateful for the financial support from the University of Vienna, the Jagiellonian University from Kraków, the University of Twente, and the Ministry of Science and Higher Education in Poland that we received in response to our requests. However, this financial support is insufficient. Therefore, our policy of publishing articles free of cost for the authors is history. As time was running out for a different solution, we decided to start charging authors an article processing fee (APF), a policy that is in effect from now on.

As our journal still receives funding from several sources, see above, we will be able to keep these costs at a relatively low level. We will start with an APF of 300,- € and will see how far this gets us. Of course, contributions by authors affiliated to institutions funding ACP as well as contributions authored by the members of our Editorial Board will be waived and continue to be free of cost. We hope that this policy also encourages further institutional financial contributions because such support will now correspond to a flat rate for the publications of their affiliated authors. This also means that, if you want, each of you can ask any institutions for financial support of ACP that you think might sensibly be interested in supporting our journal. Eventually, we might thus be able to return to a free-of-cost publishing policy in the future. As originally estimated, annual support of 1000,- € by about ten institutions would suffice.

Another relevant development is the decision to apply the creative commons license (see https://creativecommons.org) to articles published in our journal. This policy has been implemented from the start of this year. We chose the CC-BY-NC-ND license. This implies that people are free to copy and distribute articles if they give appropriate credits to the authors. However, the articles published in ACP may not be used for commercial purposes, and it is not allowed to change ACP articles and distribute modified ACP articles. This new policy implies that our journal fulfills the latest requirements for Open Access journals, as specified by the directory of open access journals (https://doaj.org). As a consequence of our creative commons license, university libraries are allowed to keep copies or links to articles published in ACP, which up to now was not stated clearly.

We also welcomed a new editor. Since the end of November 2016, Peter Wühr from the Technical University of Dortmund serves as an action editor and editorial member for ACP. Peter will take care of manuscripts within his field of expertise. Peter’s research focuses on attention, action control, conflict adaptation, dual-task performance, working memory and perception-action interactions. He is also interested in more applied topics, such as judging offside in soccer, the effects of musical training on cognitive abilities, and the psychometric assessment of attention.

## Performance of the journal in 2016

First of all, in 2016, we received a second Impact Factor (1.444). Secondly, out of 89 submissions in 2016, 26% were rejected without external review, 39% were rejected after external review, 12% were accepted, and the remaining manuscripts are currently being revised or are under review.

In conclusion of this letter, we wanted to express our gratitude to the reviewers that we consulted in 2016: You played a key role in keeping our high standards, and special thanks go to you! Your names can also be found on our website. Finally, we kindly wanted to remind you that we continue to welcome your submissions to the journal. Should you have any questions or comments regarding this letter or ACP in general, please feel always free to contact us.

Sincerely yours,

Rob van der Lubbe Co-editor in chief

Ulrich Ansorge Editor in chief

